# Maternal Vitamin C and Iron Intake during Pregnancy and the Risk of Islet Autoimmunity and Type 1 Diabetes in Children: A Birth Cohort Study

**DOI:** 10.3390/nu13030928

**Published:** 2021-03-13

**Authors:** Markus Mattila, Leena Hakola, Sari Niinistö, Heli Tapanainen, Hanna-Mari Takkinen, Suvi Ahonen, Jorma Ilonen, Jorma Toppari, Riitta Veijola, Mikael Knip, Suvi M. Virtanen

**Affiliations:** 1 Unit of Health Sciences, Faculty of Social Sciences, Tampere University, FI-33014 Tampere, Finland; leena.hakola@tuni.fi (L.H.); hanna-mari.takkinen@tuni.fi (H.-M.T.); suvi.ahonen@tuni.fi (S.A.); suvi.virtanen@thl.fi (S.M.V.); 2 Research, Development and Innovation Center, Tampere University Hospital, P.O. Box 2000, FI-33521 Tampere, Finland; 3 Health and Well-Being Promotion Unit, Finnish Institute for Health and Welfare, P.O. Box 30, FI-00271 Helsinki, Finland; sari.niinisto@thl.fi; 4 Population Health Unit, Finnish Institute for Health and Welfare, P.O. Box 30, FI-00271 Helsinki, Finland; heli.tapanainen@thl.fi; 5 Immunogenetics Laboratory, Institute of Biomedicine, University of Turku, FI-20014 Turku, Finland; jsilonen@utu.fi; 6 Research Centre for Integrative Physiology and Pharmacology, Institute of Biomedicine, University of Turku, FI-20520 Turku, Finland; jortop@utu.fi; 7 Department of Pediatrics, Turku University Hospital, FI-20520 Turku, Finland; 8 PEDEGO Research Unit, Department of Pediatrics, Medical Research Center, University of Oulu, P.O. Box 8000, FI-90014 Oulu, Finland; riitta.veijola@oulu.fi; 9 Department of Children and Adolescents, Oulu University Hospital, P.O. Box 10, FI-90029 Oulu, Finland; 10 Pediatric Research Center, Children’s Hospital, University of Helsinki and Helsinki University Hospital, FI-00029 Helsinki, Finland; mikael.knip@helsinki.fi; 11 Folkhälsan Research Center, FI-00251 Helsinki, Finland; 12 Research Program for Clinical and Molecular Metabolism, Faculty of Medicine, University of Helsinki, FI-00014 Helsinki, Finland; 13 Department of Pediatrics, Tampere University Hospital, FI-33521 Tampere, Finland; 14 Center for Child Health Research, Tampere University and Tampere University Hospital, FI-33014 Tampere, Finland

**Keywords:** pregnancy, nutrition, vitamin C, ascorbic acid, iron, islet autoimmunity, type 1 diabetes, birth cohort

## Abstract

Our aim was to study the associations between maternal vitamin C and iron intake during pregnancy and the offspring’s risk of developing islet autoimmunity and type 1 diabetes. The study was a part of the Finnish Type 1 Diabetes Prediction and Prevention (DIPP) prospective birth cohort including children genetically at risk of type 1 diabetes born between 1997–2004. The diets of 4879 mothers in late pregnancy were assessed with a validated food frequency questionnaire. The outcomes were islet autoimmunity and type 1 diabetes. Cox proportional hazards regression analysis adjusted for energy, family history of diabetes, human leukocyte antigen (HLA) genotype and sex was used for statistical analyses. Total intake of vitamin C or iron from food and supplements was not associated with the risk of islet autoimmunity (vitamin C: HR 0.91: 95% CI (0.80, 1.03), iron: 0.98 (0.87, 1.10)) or type 1 diabetes (vitamin C: 1.01 (0.87, 1.17), iron: 0.92 (0.78, 1.08)), neither was the use of vitamin C or iron supplements associated with the outcomes. In conclusion, no association was found between maternal vitamin C or iron intake during pregnancy and the risk of islet autoimmunity or type 1 diabetes in the offspring.

## 1. Introduction

Dietary factors during the fetal period, infancy and childhood are implicated to trigger, inhibit or modify the autoimmune processes leading to type 1 diabetes [1]. Vitamin C (ascorbic acid) and iron are essential micronutrients that human body cannot produce, and therefore, they are needed from the diet [2,3]. Vitamin C might have a protective role against type 1 diabetes due to its antioxidant properties [4,5]. In contrast, an excessive amount of iron might lead to the generation of oxygen radicals and increased inflammation and, thus, increase the risk of type 1 diabetes [3,6]. However, prospective studies assessing these nutrients in the disease process of type 1 diabetes are scarce.

In two retrospective case-control studies, the child’s intake of dietary vitamin C was not associated with type 1 diabetes [7,8], whereas in one study, dietary vitamin C supplementation was associated with decreased risk of type 1 diabetes [9]. A recent study conducted by The Environmental Determinants of Diabetes in the Young (TEDDY) Study group suggested that high plasma ascorbic acid status during childhood could decrease the risk of islet autoimmunity [10]. The maternal intake of vitamin C during pregnancy and the risk of islet autoimmunity have so far been analyzed only in the Finnish Type 1 Diabetes Prediction and Prevention (DIPP) Nutrition Study by us [11]. The intake of vitamin C from diet only or from diet and vitamin C supplementation combined was not associated with the risk of developing islet autoimmunity. We or, to our knowledge, others have not reported the association of maternal vitamin C intake with clinical type 1 diabetes before.

High cord blood iron concentration [12] and maternal use of iron supplementation during pregnancy [13] have been linked to increased risk of type 1 diabetes. Mechanisms are not yet known, but iron overload was suggested to generate reactive oxygen species that might lead to beta-cell apoptosis or ferroptosis, i.e., cell death depended on iron [3]. Beta cells are lacking sufficient antioxidant enzymes that are available in other tissues [14,15]. Vitamin C enhances the absorption of non-heme iron from diet [16]. Furthermore, in pregnant women, the use of vitamin C supplements in addition to iron supplements has increased maternal plasma iron status [17]. However, the interaction between vitamin C and iron intake on the risk of type 1 diabetes development has not been studied previously. 

Our aim was to study the associations between maternal intake of vitamin C and iron during pregnancy as well as use of dietary supplements with vitamin C or iron on the risk of islet autoimmunity and type 1 diabetes in a prospective birth cohort. We hypothesized that high maternal vitamin C intake is associated with decreased risk, while high iron intake is associated with increased risk of islet autoimmunity and type 1 diabetes in the offspring. Since vitamin C enhances the absorption of iron, we also studied interaction between maternal iron and vitamin C intake on the risk of outcomes.

## 2. Materials and Methods 

The DIPP Study is a large population-based birth cohort study of children with human leukocyte antigen (HLA)-conferred genetic risk of type 1 diabetes [18]. In the DIPP Nutrition Study within the DIPP cohort, 7782 children born in the Tampere and Oulu University Hospitals between October 1997 and September 2004 were invited for follow-up. Children with the genotypes HLA-DQB1*02/*03:02 and DQB1*03:02/x (x stands for alleles other than DQB1*02 or DQB1*06:02) were eligible for the follow-up. At the time of screening, 99% of the Finnish population was of ethnic Finnish origin. Migrant children with parents that could not speak either Finnish or Swedish and those with severe diseases or anomalies were excluded. The children were invited to study visits at the age of 3, 6, 12, 18 and 24 months and then annually up to the age of 15 years or until type 1 diabetes diagnosis. The visit interval of autoantibody positive children was 3 months. In the current report, the inclusion criteria for the analyses was the available maternal dietary assessment. Parents gave written informed consent for both genetic testing of their newborn infant from cord blood sample and for participation in the follow-up. The study adheres to the Declaration of Helsinki, and the ethics committees of Oulu and Tampere University Hospitals approved the study protocol (ETL 97193M).

### 2.1. Maternal Vitamin C and Iron Intake

The mothers were asked to report their diet during pregnancy with a validated 181-item semi-quantitative food frequency questionnaire (FFQ) [19]. These pregnancy FFQs were sent to the mothers after delivery and checked at the 3-month follow-up visit. Mothers were asked to answer retrospectively about their diet during the eighth month of pregnancy (the last month preceding maternity leave in Finland) [19]. The frequency (not at all, number of times per month, week or day) and the amounts (units of common serving sizes) of consumed foods were inquired. General units were used for some foods such as eggs and beverages. Mothers were also asked about the use of nutritional supplements over the whole time of pregnancy. The name of supplement, the manufacturer and the dosage per day, week or month were asked. The vitamin C and iron intake from vitamin C and iron only supplements and multivitamin supplements were combined in the calculation. Individual vitamin C and iron intakes were calculated based on the Finnish Food Composition Database (Fineli) using the in-house software (Finessi) of the Finnish Institute for Health and Welfare, Finland [20]. The nutrient intakes were calculated from unprocessed vegetables and fruits, which does not consider the loss of vitamin C due to processing and cooking. Energy from dietary fiber was included in the total energy. FFQs with >10 missing items were excluded. Any implausible values were double checked on the original FFQ and from the database.

### 2.2. Definiton of Type 1 Diabetes-Related Outcomes

Children were screened for islet cell autoantibodies (ICA) at intervals of 3–12 months as described before [18]. When participant had seroconversion for ICA for the first time, all of the previous and subsequent samples were analyzed for insulin autoantibodies (IAA), glutamic acid decarboxylase antibodies (GADA) and islet antigen-2 antibodies (IA-2A). ICA was quantified by a standard indirect immunofluorescence method, IAA, GADA and IA-2A with specific radiobinding assays. Transplacentally transferred autoantibodies were not considered as the child’s endogenous autoantibodies. The definition for islet autoimmunity was repeated positively for ICA and at least one biochemical autoantibody (IAA, GADA, IA-2A) or having type 1 diabetes (one child was diagnosed with type 1 diabetes without information on autoantibody positivity). Data on the diagnosis of type 1 diabetes were obtained in May 2015 from Finnish Pediatric Diabetes Register and University Hospitals. The diagnosis of type 1 diabetes was defined according to World Health Organization criteria [21]. In the present study, children were considered free from type 1 diabetes if they were not found in the register. Of the children invited, two datasets were formed. The islet autoimmunity cohort included 4887 children, and the type 1 diabetes cohort included 4943 children. Data on maternal diet during pregnancy were available for 4879 pregnancies, as 64 mothers had twin pregnancies. The mothers who were invited to the study but had insufficient data on maternal diet were more likely to be in the lowest or highest age category, more likely to be smokers and had more previous deliveries and a less education than those mothers with dietary data [22].

### 2.3. Genetic Methods

HLA-DQ was genotyped using panels of sequence-specific oligonucleotide probes, as described before [18]. Genotypes HLA-DQB1 (*02/*03:02) represent “high” and HLA-DQB1*03:02/x (x ≠ *02, *03:01, *06:02) “moderate” risk for type 1 diabetes.

### 2.4. Background Characteristics

Information on maternal education, diabetes (unspecified type) and family history of diabetes among the first-degree relatives was collected with a questionnaire after the delivery. Information on the offspring’s sex, maternal age and maternal smoking during pregnancy was obtained from the birth registers of the hospitals. Maternal BMI was determined from the mother’s weight at the first prenatal visit as described previously [23]. 

### 2.5. Statistical Methods

Differences in maternal vitamin C and iron intake (in mg/MJ) between groups of potentially confounding background characteristics were tested using one-way ANOVA. Differences in characteristics between supplement users and non-users were tested using t-test and Pearson Chi-square test. In the analyses exploring the risk of islet autoimmunity and type 1 diabetes, maternal vitamin C and iron intakes from diet and total intake (including the intake from supplements) were energy-adjusted using Willett’s residual method [24]. Maternal vitamin C and iron intake were analyzed both as continuous and categorized variables. The intakes were categorized into quartiles, from which the combined two middle quartiles were used as the reference category. The use of supplements with vitamin C and iron at any time during pregnancy was categorized as yes/no. The risk of islet autoimmunity and type 1 diabetes was assessed with Cox proportional hazards regression analysis.

The main analyses were energy-adjusted with the Willett’s residual method and further adjusted for sex (boy or girl), family history of diabetes (yes or no) and HLA genotype (high or moderate risk). Another model was also adjusted for maternal education, pre-pregnancy BMI and smoking.

We tested whether child’s sex modified the association between the vitamin C and iron intake and the outcomes. The results indicated no interaction, and therefore, main analyses included girls and boys together. To test whether total vitamin C intake modified the association between total iron intake and the outcomes, an interaction term was added into the model.

SAS software version 9.3 (SAS Institute, Cary, NC, USA) and IBM SPSS Statistics version 25.0 (IBM Corporation, Armonk, NY, USA) were used in the analyses. Statistical significance was set at 2-sided *p* < 0.05. 

## 3. Results

### 3.1. Background Characteristics

Altogether 312 (6.4%) children developed a positive islet autoimmunity at a median age of 3.5 (IQR 1.7–6.6) years, and 178 (3.6%) had type 1 diabetes at the median age of 7.1 (IQR 4.3–10.6) years during the 15-year follow-up. The dropout rates among the 4887 children at 1- and 5-year autoantibody follow-up were 5.7% (279 children) and 30% (1415 children), respectively. 

Maternal total vitamin C and iron intake by background variables are presented in Table 1. Mothers with lower education had lower vitamin C intake during pregnancy than those with higher education (Table 1). Mothers who smoked had lower iron intake than non-smokers. Lower maternal BMI in early pregnancy was associated with higher iron intake and non-linearly with vitamin C and iron intake (Table 1). 

Mothers who used supplementation with vitamin C (1555 mothers, 32%) had lower BMI (23.8 vs. 24.5 kg/m^2^, *p* < 0.001), higher education (overall *p* < 0.001), were more often non-smokers (92 vs. 89%, *p* = 0.001) and had a higher intake of dietary iron (16.9 vs. 16.5 mg/day, *p* < 0.001) compared to those who did not use supplementation with vitamin C. 

Mothers who used iron supplementation during pregnancy (3375 mothers, 69%) had lower BMI (23.9 vs. 25.2 kg/m^2^, *p* < 0.001), were more often highly educated (overall *p* < 0.001) and non-smokers (92% vs. 86%, *p* < 0.001) and had a higher total energy (11.8 vs. 11.5 MJ/day, *p* = 0.01) and higher iron intake from food (16.2 vs. 15.8 mg/day, *p* < 0.001), in comparison to those who did not use iron supplementation.

### 3.2. Intake and Dietary Sources of Vitamin C and Iron

Mean (SD) maternal intake of vitamin C from foods during pregnancy was 198 (116) mg/day and from supplements 23 (82) mg/day. The most important sources of vitamin C were juices, vegetables, fruits, and dietary supplements (Table 2). A total of 240 (5%) of the mothers consumed less than 70 mg/day of vitamin C, which was the recommended intake of pregnant mothers in Finland during the time of dietary assessment. Only five (0.1%) mothers had insufficient vitamin C intake < 20 mg/day.

Mean (SD) maternal intake of iron from foods during pregnancy was 17 (5) mg/day and from supplements 26 (33) mg/day. Dietary supplements, cereals and meats were the main source of iron (Table 2).

### 3.3. Maternal Vitamin C and Iron Intake and Risk of Type 1 Diabetes-Realted Outcomes

Energy-adjusted maternal intake of vitamin C or iron from food and total intake (from food and supplements together) during pregnancy were not associated with islet autoimmunity or type 1 diabetes (Table 3). Results were similar for both continuous and categorized intake. Further adjustment for sex, family history of diabetes and HLA genotype did not change the risk significantly (Table 3) nor did the adjustment for maternal education, pre-pregnancy BMI and smoking (Supplementary Table S1). The maternal use vs. non-use of dietary supplements with vitamin C during pregnancy was not associated with the risk of islet autoimmunity (HR 1.08 (95% CI 0.86, 1.37), *p* = 0.51) or type 1 diabetes (1.18 (0.87, 1.61), *p* = 0.28). Adjustment for sex, family history of diabetes and HLA genotype did not change the results for islet autoimmunity (1.05 (0.83, 1.33), *p* = 0.67) or type 1 diabetes (1.14 (0.84, 1.56), *p* = 0.39). Further adjustment for maternal education, pre-pregnancy BMI and smoking did not change the results for islet autoimmunity (1.07 (0.83, 1.36), *p* = 0.61) or type 1 diabetes (1.14 (0.82, 1.58), *p* = 0.43). Maternal use of supplements with iron during pregnancy was not associated with the risk of islet autoimmunity (1.18 (0.91, 1.51), *p* = 0.21) or type 1 diabetes (1.17 (0.84, 1.62), *p* = 0.36). Adjustment for sex, family history of diabetes and HLA genotype did not change the results for islet autoimmunity (1.11 (0.86, 1.43), *p* = 0.43) or type 1 diabetes (1.14 (0.82, 1.58), *p* = 0.45). Further adjustment for maternal education, pre-pregnancy BMI and smoking did not change the results for islet autoimmunity (1.20 (0.91, 1.58), *p* = 0.20) or type 1 diabetes (1.18 (0.82, 1.69), *p* = 0.37).

### 3.4. Interaction Analyses

We observed no interaction between energy-adjusted total vitamin C intake and total iron intake on the risk of islet autoimmunity or type 1 diabetes.

## 4. Discussion

In this large prospective birth cohort of children with increased genetic risk for type 1 diabetes, the maternal intake of vitamin C or iron during pregnancy and use of supplementation with vitamin C or iron were not associated with the risk of islet autoimmunity or type 1 diabetes.

The strengths of this study include a large study population, carefully collected data on maternal dietary intake and use of supplementation, use of well-maintained food composition database, as well as regular assessment of autoantibodies and type 1 diabetes. In the validation study of the FFQ used in our study, the FFQ in comparison to food records (10 days) showed a correlation of 0.65 for vitamin C and 0.60 for iron [19], suggesting that the FFQ is appropriate for the estimation of vitamin C and iron intake. Furthermore, no previous studies have explored the association between both dietary and supplementary intake of iron and the risk of islet autoimmunity.

Our study also had some limitations. The validation study of the FFQ did not include the use of dietary supplements [19]. Furthermore, food storage and processing can affect the vitamin C content in the foods, which cannot be totally taken into account in the food composition databases. Another limitation is that the vitamin C intake is linked to fruit and vegetable consumption, which may be confounded by several socioeconomic and lifestyle factors [25]. Additionally, we did not have plasma vitamin C and iron status available in our study, which could have provided us with an additional and, likely, a more accurate biomarker of these nutrients in the body [25,26].

Our results do not support the hypothesis that higher intake of vitamin C during pregnancy would protect from islet autoimmunity or type 1 diabetes. As far as we know, this is the only prospective study so far to explore the maternal intake of vitamin C and the risk of clinical type 1 diabetes outcome. In an earlier study with fewer mothers and children, we explored the association between maternal vitamin C intake and islet autoimmunity outcome [11]. Results were similar than in the present study but the use of vitamin C supplements per se were not studied in the previous study. The current study was carried out in a well-nourished population, and the mean vitamin C intake in mothers was more than triple the amount of the recommended 70 mg/day during pregnancy [27]. Only 240 (5%) mothers had an intake below the recommended intake, and only five (0.1%) mothers had a vitamin C intake under 20 mg/day in which plasma ascorbic acid status begins to deteriorate [28]. However, the FFQ in our study is likely to overestimate the intake of foods, which are considerable sources of vitamin C such as vegetables and fruits [19]. In addition, the same maternal characteristics were associated with non-participation and lower intakes of vitamin C, suggesting the final study population may have a higher intake of vitamin C compared to those who did not participate in the follow-up. Therefore, we cannot conclude whether vitamin C would be associated with risk of islet autoimmunity or type 1 diabetes in populations with lower vitamin C intakes. Our study did not include plasma ascorbic acid status, which is suggested to represent vitamin C function more accurately than dietary intake since absorption, transport and physiological requirements of vitamin C vary between individuals [2,25]. Plasma ascorbic acid status should be considered in future studies as high plasma ascorbic acid status in childhood has been associated with reduced risk of islet autoimmunity in a previous study [10].

Our results do not support the hypothesis that high maternal intake of iron from the diet or supplements would increase the risk of islet autoimmunity or type 1 diabetes in the offspring. Two previous prospective studies exploring maternal iron supplement use and the risk of clinical type 1 diabetes gave inconsistent results. While the Danish National Birth Cohort Study found no association between maternal iron supplement use during pregnancy and type 1 diabetes in the offspring [29], the Norwegian Mother and Child Cohort Study observed that maternal use of any iron-containing supplements was associated with increased risk of type 1 diabetes [13]. Mothers in our study and previous studies were from well-nourished populations and the use of iron supplements was common. The Finnish dietary recommendation states that iron supplementation may be needed after the first trimester to maintain an iron balance of 500 mg iron storage [27,30]. Hence, some of the mothers in our study may have used supplements without any actual need. Similarly to the Norwegian cohort study, iron intake from food was not associated with type 1 diabetes risk in the current report [13].

Although we did not find association between maternal iron intake type 1 diabetes development, iron is an important nutrient for further studies. Mechanisms between maternal iron exposure and fetal outcomes might be complex. Maternal use of iron supplement during pregnancy may induce oxidative stress in the placenta, but the implication for the offspring has yet to be explored [13,31]. Maternal iron bioavailability is regulated by hepcidin, which suppresses the excess maternal iron flow in the circulation when iron supplements are taken regularly [32,33]. Fetal hepcidin and iron transport proteins in the placenta might also protect fetuses from iron overload [33]. In addition, epigenetic mechanisms may play an important role since, e.g., maternal hemochromatosis gene (HFE) genotypes have been associated with increased risk of type 1 diabetes [13,34]. Polymorphisms in the HFE gene induces hereditary hemochromatosis and increased iron stores, which could cause iron accumulation in the endocrine pancreas and beta-cell injury [35,36].

## 5. Conclusions

In conclusion, maternal vitamin C or iron intake during pregnancy were not associated with the risk of developing islet autoimmunity or type 1 diabetes in a prospective cohort of children carrying increased genetic susceptibility to type 1 diabetes.

## Figures and Tables

**Table 1:** Characteristics of 4879 mothers in relation to mean (standard deviation) total intake of vitamin C during and iron 8th month of pregnancy.

	Maternal Intake during Pregnancy		
		Vitamin C, mg/MJ	Iron, mg/MJ
Characteristic	*n*	Mean (SD)	Mean (SD)
Maternal age, years			
≤24	926	18.7 (11.1)	3.8 (3.4)
25–29.9	1700	18.8 (10.3)	3.7 (3.0)
30–34.9	1412	19.3 (13.4)	3.9 (3.2)
≥35	841	19.1 (12.2)	3.8 (2.9)
*p*-value ^b^		0.46	0.72
Maternal BMI in early pregnancy, kg/m^2^			
<25	3025	19.3 (11.9)	4.0 (3.3)
25–29.9	1123	18.1 (10.5)	3.6 (2.9)
≥30	434	18.7 (11.1)	3.4 (2.9)
Missing	297		
*p*-value ^a^		0.01	<0.001
Maternal weight gain rate, kg/week ^c^			
1st quarter < 0.33	1136	19.4 (13.6)	3.9 (3.2)
2nd quarter 0.33–0.41	1137	18.4 (9.8)	3.9 (3.3)
3rd quarter 0.42–0.52	1137	18.8 (10.9)	3.8 (3.0)
4th quarter > 0.52	1136	19.1 (11.3)	3.8 (3.1)
Missing	333		
*p*-value ^a^		0.14	0.48
Maternal vocational education ^d^			
None	294	18.2 (13.5)	3.6 (3.2)
Vocational School or Course	1291	18.4 (11.5)	3.9 (3.5)
Secondary Vocational Education	2067	18.9 (11.7)	3.8 (3.0)
University Studies or Degree	1097	20.1 (11.8)	3.8 (3.1)
Missing	130		
*p* value ^a^		0.001	0.40
Maternal smoking during pregnancy			
Yes	467	18.1 (12.7)	3.5 (3.6)
No	4246	19.1 (11.7)	3.9 (3.1)
Missing	166		
*p* value ^a^		0.10	0.04
Maternal diabetes ^d^			
Yes	164	19.0 (11.9)	4.0 (3.1)
No	4611	19.0 (11.8)	3.8 (3.1)
Missing	104		
*p* value ^a^		0.89	0.49

^a^ total intake based on diet and dietary supplements during 8th month of pregnancy. ^b^ *p* values for difference between groups from one-factor ANOVA. ^c^ At the time of birth. ^d^ Based on a questionnaire completed after birth. Type of diabetes not specified.

**Table 2:** Maternal intake of vitamin C and iron during pregnancy from food groups, DIPP Nutrition Study, Finland.

Vitamin C and Iron Intake from Food Groups	Mean Vitamin C mg/day	% of Intake	Mean Iron mg/day	% Of Intake
Fruits and berries ^1^	41.0	18.7	0.69	1.6
Fruit juices	84.8	38.6	0.28	0.6
Other sweetened fruit drinks	2.1	1.0	0.32	0.7
Vegetables	46.5	21.2	1.14	2.7
Leaf vegetables	3.4	1.6	0.19	0.5
Fruit vegetables	20.9	9.5	0.31	0.7
Root vegetables	8.7	4.0	0.29	0.7
Other vegetables ^2^	13.4	6.1	0.35	0.8
Legumes, nuts, seeds and soy products	0.8	0.4	0.32	0.8
Potatoes and potato-based products	10.3	4.7	0.83	1.9
Dairy products	9.7	4.4	0.54	1.3
Cereals	0.01	0	6.52	15.3
Egg and egg dishes	0	0	0.70	1.6
Fish and fish dishes	0	0	0.23	0.5
Meat and meat dishes (beef, pork, lamb, poultry, game)	0.5	0.2	3.42	8.1
Unprocessed meat	0	0	1.64	3.9
Processed meat ^3^	0.5	0.2	1.78	4.2
Other foods ^4^	0.6	0.3	1.21	2.8
Dietary supplements ^5^	23.1	10.5	26.31	61.9
Total	219.4	100	42.50	100

^1^ Including canned and dried fruits and berries. ^2^ Cabbages, onions, mushrooms and canned vegetables. ^3^ Sausages and cured meat products. ^4^ Fats, oils, beverages, sugars, sweets and condiments. ^5^ Contains vitamin C or iron exclusively supplements and multivitamins.

**Table 3:** Hazard ratios (HR) and 95% confidence intervals (95% CI) for the association between maternal intake of vitamin C and iron during pregnancy and the risk of islet autoimmunity and type 1 diabetes in the offspring.

	Islet Autoimmunity				Type 1 Diabetes			
	Model 1 *n* = 4887 (312) ^a^		Model 2 *n =* 4706 (305) ^a^		Model 1 *n* = 4943 (178) ^a^		Model 2 *n =* 4757 (174) ^a^	
	HR ^b^ (95% CI)	*p*-Value	HR ^b^ (95% CI)	*p*-Value	HR ^b^ (95% CI)	*p*-Value	HR ^b^ (95% CI)	*p*-Value
Vitamin C from diet								
per 1 SD increase	0.97 (0.87, 1.09)	0.62	0.98 (0.87, 1.10)	0.73	1.05 (0.91, 1.21)	0.51	1.07 (0.93, 1.23)	0.37
Q_1_	0.96 (0.73, 1.27)	0.96	0.99 (0.75, 1.30)	0.98	1.10 (0.77, 1.58)	0.47	1.08 (0.75, 1.57)	0.41
Q_2_ and Q_3_	1 (ref)		1 (ref)		1 (ref)		1 (ref)	
Q_4_	0.98 (0.75, 1.29)		1.02 (0.77, 1.34)		1.25 (0.88, 1.77)		1.27 (0.89, 1.81)	
Total vitamin C intake ^c^								
per 1 SD increase	0.90 (0.79, 1.03)	0.12	0.91 (0.80, 1.03)	0.14	0.99 (0.86, 1.15)	0.94	1.01 (0.87, 1.17)	0.92
Q_1_	0.96 (0.74, 1.26)	0.32	0.98 (0.75, 1.28)	0.36	0.92 (0.64, 1.32)	0.74	0.88 (0.61, 1.28)	0.73
Q_2_ and Q_3_	1 (ref)		1 (ref)		1 (ref)		1 (ref)	
Q_4_	0.80 (0.61, 1.07)		0.82 (0.61, 1.09)		0.87 (0.60, 1.26)		0.89 (0.61, 1.29)	
Iron from diet								
per 1 SD increase	1.09 (0.99, 1.22)	0.09	1.07 (0.96, 1.19)	0.22	1.10 (0.96, 1.26)	0.17	1.09 (0.95, 1.25)	0.23
Q_1_	0.70 (0.52, 0.94)	0.06	0.71 (0.53, 0.97)	0.09	0.63 (0.42, 0.94)	0.06	0.95 (0.72, 1.26)	0.71
Q_2_ and Q_3_	1 (ref)		1 (ref)		1 (ref)		1 (ref)	
Q_4_	0.94 (0.73, 1.23)		0.92 (0.70, 1.20)		1.00 (0.71, 1.40)		0.89 (0.68, 1.18)	
Total iron intake ^c^								
per 1 SD increase	0.99 (0.88, 1.11)	0.82	0.98 (0.87, 1.10)	0.71	0.94 (0.81, 1.10)	0.46	0.92 (0.78, 1.08)	0.30
Q_1_	0.94 (0.71, 1.24)	0.79	0.95 (0.72, 1.26)	0.71	1.01 (0.71, 1.45)	0.98	1.01 (0.70, 1.45)	0.89
Q_2_ and Q_3_	1 (ref)		1 (ref)		1 (ref)		1 (ref)	
Q_4_	0.92 (0.70, 1.20)		0.89 (0.68, 1.18)		0.97 (0.68, 1.39)		0.92 (0.64, 1.33)	

Abbreviations: Q1–Q4, quarter. ^a^ n represents total number in the analyses and number in parenthesis represents numbers of children with the outcome. ^b^ HRs (95% CI) are from Cox regression analysis (*p* values from Wald test). ^c^ Total vitamin C and total iron intake from diet and dietary supplements. Model 1: Energy adjusted with the Willett’s residual method. Model 2: As model 1 and further adjusted for sex, family history of diabetes HLA genotype.

## Data Availability

The data is available from the corresponding author on reasonable request. The data are not publicly available due to the protection of the identity of the study participants and their clinical data.

## Supplementary Materials

The following are available online at https://www.mdpi.com/2072-6643/13/3/928/s1, Table S1: Maternal intake of vitamin C and iron during pregnancy and hazard ratios (HR) and 95% confidence intervals (95% CI) for islet autoimmunity and type 1 diabetes in the offspring, additional adjustments.
