# Supplementary material for: Maternal Vitamin C and Iron Intake during Pregnancy and the Risk of Islet Autoimmunity and Type 1 Diabetes in Children: A Birth Cohort Study
Source: Nutrients. 2021 Mar 13;13(3):928. doi: 10.3390/nu13030928 (PMC8001228; doi:10.3390/nu13030928)
Supplement: Supplementary file 1 [file nutrients-13-00928-s001.pdf]

Supplementary Table S1. Maternal intake of vitamin C and iron during pregnancy and hazard ratios (HR) and 95% confidence intervals (95% CI) for islet autoimmunity and type 1 diabetes in the offspring, additional adjustments.

	**Islet Autoimmunity**		**Type 1 Diabetes**	
	**Model 3** *n* = 4253 (284)[a]		**Model 3** *n* = 4301 (160)[a]	
	**HR[b] (95% CI)**	***P*-value**	**HR[b] (95% CI)**	***P*-value**
Vitamin C from diet				
per 1 SD increase	0.95 (0.84, 1.07)	0.42	1.03 (0.88, 1.20)	0.73
Q_1	1.00 (0.75, 1.34)	0.96	1.22 (0.83, 1.79)	0.44
Q_2 and Q_3	1 (ref)		1 (ref)	
Q_4	0.96 (0.72, 1.29)		1.23 (0.84, 1.79)	
Total vitamin C intake[c]				
per 1 SD increase	0.89 (0.77, 1.02)	0.10	0.81 (0.83, 1.03)	0.15
Q_1	0.98 (0.73, 1.30)	0.17	0.93 (0.63, 1.37)	0.73
Q_2 and Q_3	1 (ref)		1 (ref)	
Q_4	0.75 (0.56, 1.02)		0.76 (0.55, 1.21)	
Iron from diet				
per 1 SD increase	1.09 (0.98, 1.22)	0.11	1.09 (0.94, 1.27)	0.23
Q_1	0.75 (0.54, 1.02)	0.17	0.75 (0.49, 1.15)	0.30
Q_2 and Q_3	1 (ref)		1 (ref)	
Q_4	1.00 (0.76, 1.31)		1.09 (0.76, 1.56)	
Total iron intake[c]				
per 1 SD increase	0.99 (0.87, 1.11)	0.82	0.92 (0.78, 1.09)	0.34
Q_1	0.86 (0.64, 1.16)	0.47	0.94 (0.64, 1.40)	0.78
Q_2 and Q_3	1 (ref)		1 (ref)	
Q_4	0.87 (0.65, 1.15)		0.87 (0.59, 1.28)	

Abbreviations: Q1-4, quarter

[a] *n* represents total number in the analyses and number in parenthesis represents numbers of children with the outcome

[b] HRs (95% CI) are from Cox regression analysis (*P* values from Wald test)

[c] Total vitamin C and total iron intake from diet and dietary supplements

Model 3: Adjusted for energy residual method, sex, familial diabetes, HLA genotype, maternal education, pre-pregnancy BMI, and smoking
